# The Oxygen Treatment of Wounds

**Published:** 1897-03-06

**Authors:** 


### Editor's Letter-Box.

[Our Correspondents are reminded that prolixity is a great bar to publication, and that brevity of style and conciseness of statement greatly facilitate early insertion.]

### THE OXYGEN TREATMENT OF WOUNDS.

Mr. George Stoker writes: I beg to thank you for your fair and very opportune article in reference to the above, and to say that if the staff of any hospital wish to test my system I will be happy to give any assistance in my power. I must, however, respectfully protest against the suggestion that "the interest of the matter seems to us to lie chiefly in the bacteriology, and it is by that that the whole proceeding must stand or fall." This, like every other system, must stand or fall by its success or failure to cure, and not by the correctness or otherwise of any theory as to the changes, either chemical or bacteriological, by which success is brought about. I would further like to point out that the application of the system is not confined to chronic ulcers only. It is possibly more efficacious in recent wounds.

